# Avoiding a replication crisis in deep-learning-based bioimage analysis

**DOI:** 10.1038/s41592-021-01284-3

**Published:** 2021-10-01

**Authors:** Romain F. Laine, Ignacio Arganda-Carreras, Ricardo Henriques, Guillaume Jacquemet

**Affiliations:** 1 MRC-Laboratory for Molecular Cell Biology, University College London, London, UK; 2 The Francis Crick Institute, London, UK; 3 Computer Science and Artificial Intelligence department, University of the Basque Country (UPV/EHU), San Sebastian, Spain; 4 Ikerbasque, Basque Foundation for Science, Bilbao, Spain; 5 Donostia International Physics Center (DIPC), San Sebastian, Spain; 6 Instituto Gulbenkian de Ciência, Oeiras, Portugal; 7 Turku Bioscience Centre, University of Turku and Åbo Akademi University, 20520 Turku, Finland; 8 Åbo Akademi University, Faculty of Science and Engineering, Biosciences, 20520 Turku, Finland; 9 Turku Bioimaging, University of Turku and Åbo Akademi University, 20520 Turku, Finland

## Abstract

Deep learning algorithms are powerful tools to analyse, restore and transform bioimaging data, increasingly used in life sciences research. These approaches now outperform most other algorithms for a broad range of image analysis tasks. In particular, one of the promises of deep learning is the possibility to provide parameter-free, one-click data analysis achieving expert-level performances in a fraction of the time previously required. However, as with most new and upcoming technologies, the potential for inappropriate use is raising concerns among the biomedical research community. This perspective aims to provide a short overview of key concepts that we believe are important for researchers to consider when using deep learning for their microscopy studies. These comments are based on our own experience gained while optimising various deep learning tools for bioimage analysis and discussions with colleagues from both the developer and user community. In particular, we focus on describing how results obtained using deep learning can be validated and discuss what should, in our views, be considered when choosing a suitable tool. We also suggest what aspects of a deep learning analysis would need to be reported in publications to describe the use of such tools to guarantee that the work can be reproduced. We hope this perspective will foster further discussion between developers, image analysis specialists, users and journal editors to define adequate guidelines and ensure that this transformative technology is used appropriately.

## Introduction

Microscopy is a leading technology to gain fundamental insight for biological research. Today, a typical microscopy session may generate hundreds to thousands of images, generally requiring computational analysis to extract meaningful results from them. Over the last few years, deep learning (DL) has increasingly become one of the gold standards for high-performance microscopy image analysis ^1,2^. DL has been shown to perform a wide range of image analysis very efficiently, such as image classification ^3,4^, object detection ^5,6^, image segmentation ^7–9^, image restoration ^10,11^, super-resolution microscopy ^10,12–15^, object tracking ^16,17^, image registration ^18^ and the prediction of fluorescence images from label-free imaging modalities ^19^.

**Figure 1:**
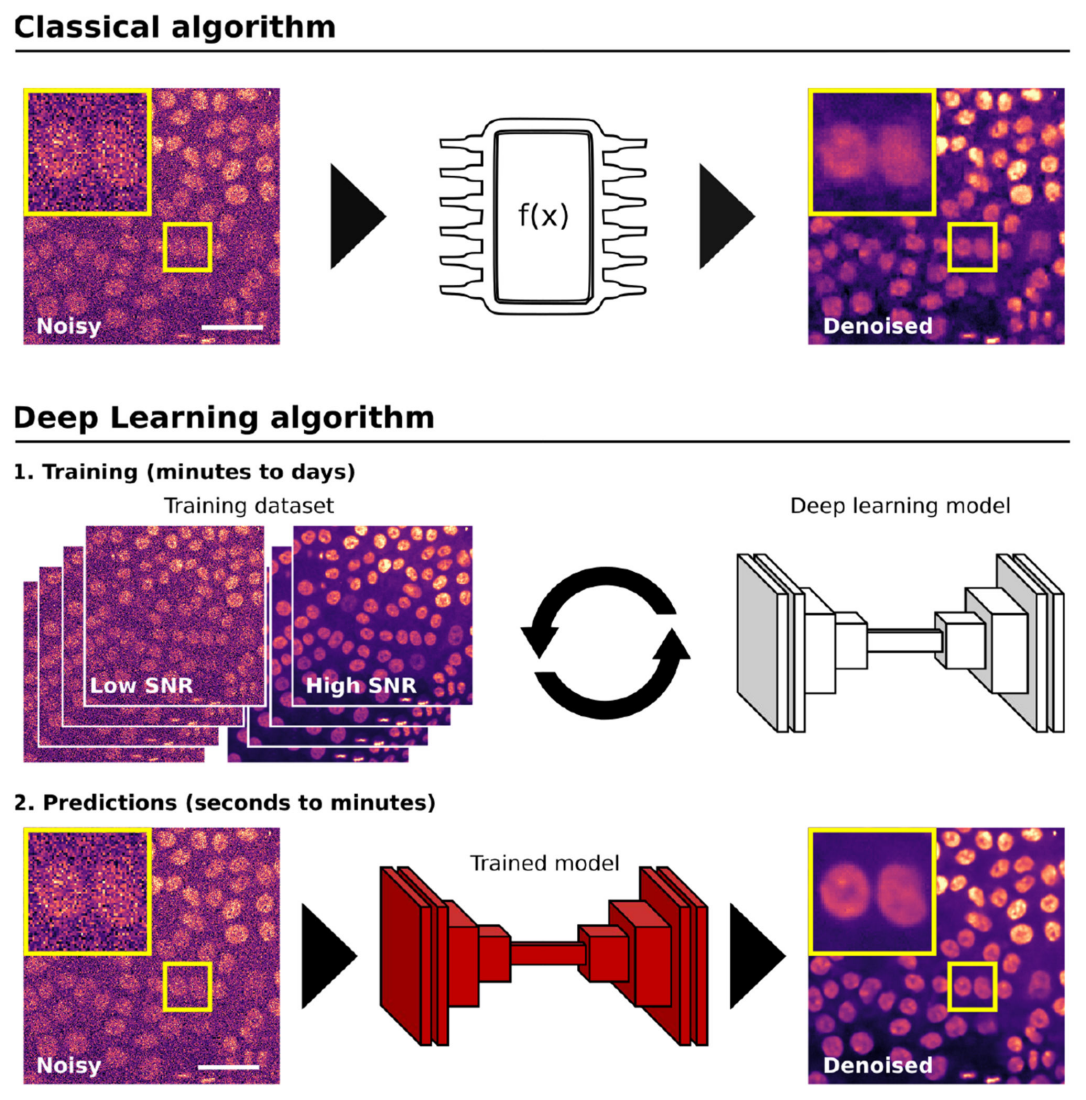
Using classical or DL algorithms to analyse microscopy images. This figure illustrates the critical steps required when using classical or DL-based algorithms to analyse microscopy images, using denoising as an example. When using a classical algorithm, the researchers’ efforts are put into designing mathematical formulae that can then be directly applied to the images. When using a DL algorithm, first, a model needs to be trained using a training dataset. Next, the model can be directly applied to other images and generate predictions. Typically, such a model will only perform well on images similar to the ones used during training. This highlights the importance of the data used to train the DL algorithm (its quantity and diversity). The microscopy images displayed are breast cancer cells labelled with SiR-DNA to visualise their nuclei and imaged using a spinning disk confocal microscope (SDCM). The denoising performed in the “classical algorithm” section was performed using PureDenoise implemented in Fiji ^20,21^. The denoising performed in the “Deep Learning algorithm” section was performed using CARE implemented in ZeroCostDL4Mic ^10,22^.

For image analysis, DL usually uses algorithms called artificial neural networks (ANNs). Unlike classical algorithms, before using an ANN, it first needs to be trained (Figure 1). During training, the ANN is presented with a range of data from which it attempts to learn how to perform a specific task (i.e. denoising). More specifically, the ANN builds a model of the mathematical transformation that needs to be applied to data to obtain the desired output. Here, the model parameters (called weights) can be seen as the instructions to carry out the learned task. Once the weights of a model are optimised, it can be used to perform the task, a step called inference or prediction. Therefore, ANNs can be considered non-linear transformation machines, performing sequential mathematical operations on the input data. As we inspect deeper into these sequences of operations, it becomes difficult to understand what features of the original images are used. For that reason, they are often thought of as “black boxes” since, for most users, only the input images and output predictions are readily available.

The training data provided to the ANN is commonly constituted of a large set of representative input images and their expected results. For instance, in denoising, the training dataset is composed of noisy and high signal-to-noise ratio (SNR) images (Figure 1). This type of training using paired image-labels is commonly referred to as supervised training. On the other hand, for so-called self-supervised training, pre-processing steps directly generate the training pairs, and therefore, the users only need to provide input images. Training is typically the most challenging, time-consuming and resource-greedy part of the process and can take minutes to weeks depending on the size of the training dataset and the type of ANN. It often requires specialised knowledge, dedicated training datasets and access to powerful computational resources such as Graphical Processing Units (GPUs) to run and optimise ANN training. In comparison, using DL models (predictions) can be straightforward (parameter-free, one-click solution) and fast (seconds to minutes). Multiple tools are in development to facilitate the training and use of DL for bioimage analysis, including both online and offline, commercial and open-source solutions ^8,22–30^.

**Figure 2:**
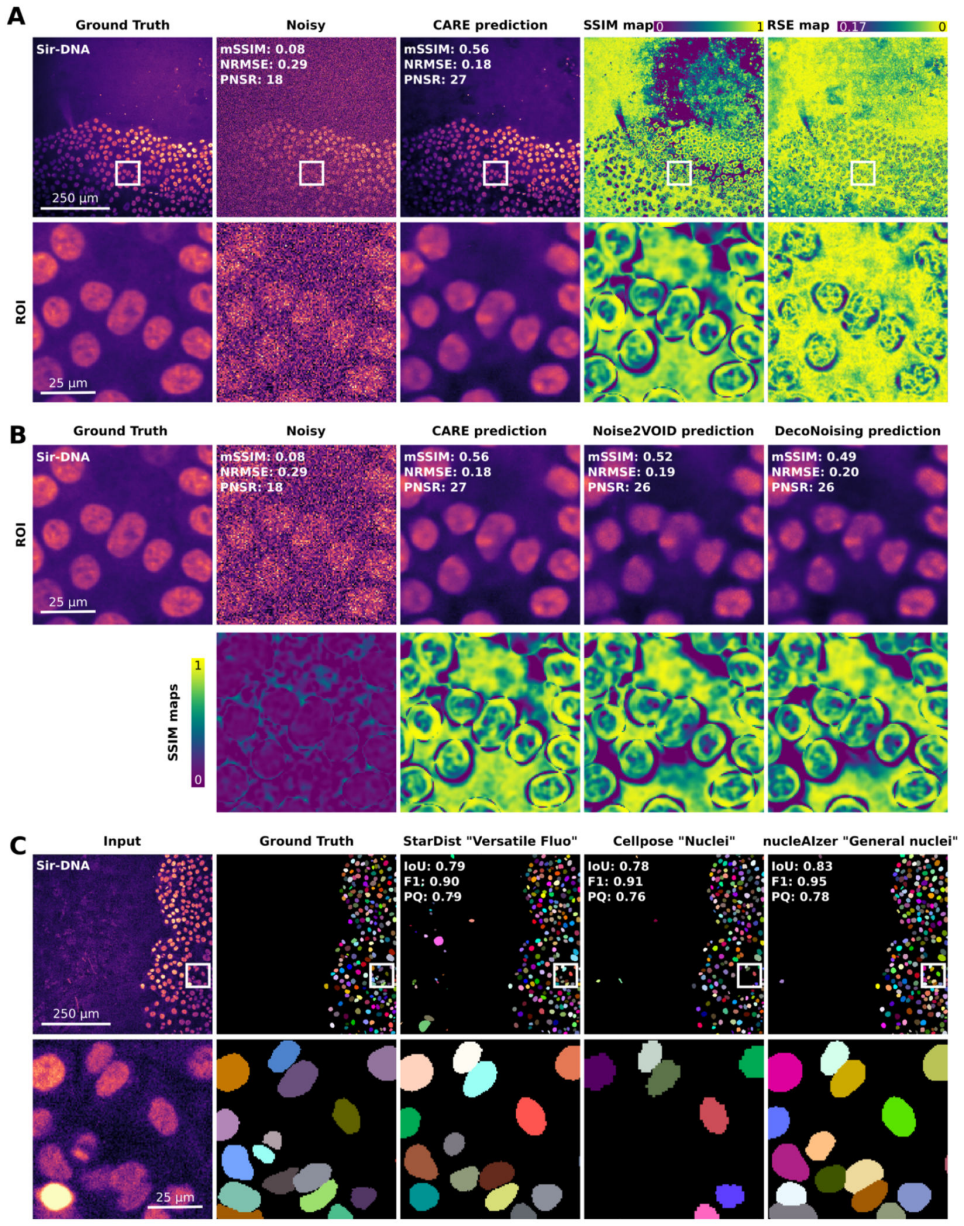
Using quality metrics to assess the performance of DL models. Figure illustrating that comparing DL-based predictions to ground truth images is a powerful strategy to assess a DL model performance. (**A**, **B**) Noisy images of breast cancer cells labelled with SiR-DNA were denoised using CARE (**A**, **B**; ^10^), Noise2Void (**B**, ^11^), and DecoNoising (**C**, ^36^) all implemented in ZeroCostDL4Mic ^22^. Noisy and ground truth images were acquired using different exposure times. (**A**) Matching noisy, ground truth, and CARE prediction images. White squares highlight regions of interest that are magnified in the bottom rows. Image similarity metrics mSSIM, NRMSE, and PNSR (see Box 1) shown on the images were obtained by comparing them to the ground truth image. The SSIM (yellow: high agreement; dark blue low agreement, 1 indicates perfect agreement) and RSE (yellow: high agreement; dark blue low agreement, 0 indicates perfect agreement) maps highlight the differences between the CARE prediction and the corresponding ground truth image. Note that the agreement between these two images is not homogenous across the field of view and that these maps are helpful to identify spatial artefacts. (**B**) Magnified region of interest from (**A**) showcasing how using image similarity metrics can compare different DL models trained using different algorithms but using the same training dataset. Note that in this example, all three algorithms improved the original image but to a different extent. Importantly, these results do not represent the algorithm’s overall performance to train these models but only assess their suitability to denoise this specific dataset. (**C**) Example highlighting how segmentation metrics can be used to evaluate the performance of segmentation pre-trained models ^29,31,32^ Image segmentation metrics Intersection over Union (loU, 1 indicates perfect agreement), F1 score (F1, 1 indicates perfect agreement), and panoptic quality (PQ, 1 indicates perfect agreement, ^37^) displayed on the images were obtained by comparing them to the ground truth image which was manually annotated. Of note, these results do not reflect the overall quality of these pre-trained models (or of the algorithm used to train them) but only assess their suitability to segment this dataset.

Once a model has been trained, it constitutes a portable algorithm to process new images, often with excellent speed performance, even on a local machine. However, in general, a DL model will only perform well on images similar to those used during training. How similar the images need to be depends on the type of network used, and aspects to consider here encompass microscope types, label types, and the SNR or optical aberrations. This highlights the importance of the data used to train the DL algorithm, both in terms of its quantity and its diversity. Therefore, one powerful approach is to produce general models with high reusability potential using a large and diverse training dataset. For example, popular nuclei or cell segmentation models have been released ^29,31,32^ (Figure 2). However, this is only possible when large heterogeneous pre-curated datasets are available, which are challenging to produce.

Nonetheless, as DL models are becoming accessible through public repositories (so-called model zoos, such as bioimage.io) or web interfaces ^29,32^, it becomes straightforward to use them directly to analyse new data. This has the advantages of speeding up DL uptake but, unless the researcher can confirm that their own data were well represented within the training dataset used initially (which can be very difficult to do), the performance of such portable models on the new data often remains unclear. One major downside of this issue is that the DL model may generate artefacts and biases that can be difficult to identify. Therefore, despite its incredible potential, the application of DL in microscopy analysis has raised concerns ^33–35^, due to a lack of transparency and understanding of its limitations, especially for generalisability. In addition to this, DL is developing at an incredible rate, which then places a significant burden on users to determine the most appropriate tools for their needs, taking into account the validity and performance of a range of approaches that are often difficult to compare.

Here, we propose that many of these concerns can be significantly alleviated by the careful assessment of DL models performance, consideration in the choice of tool and by following reporting guidelines to ensure transparency.

## Assessing DL model predictions

Currently, the most unambiguous way to assess the quality of DL model predictions is to compare them to ground truth images or labels (Figure 2A). Here we primarily focus on image restoration and segmentation tasks, but similar concepts also apply to other image-to-image DL-based image analysis. Segmentation results can be compared to manually annotated masks. In this case, expert manual annotations remain the gold standard to evaluate segmentation. Denoising results can be compared to matching high SNR images acquired with high laser power or long exposure times ^10,14^ or computationally introducing noise to high SNR data ^15^. The comparison between the model prediction and the ground truth dataset is scored using various metrics (see Box 1). These analyses are typically performed after a model has been trained. However, DL models are often evaluated using data that are similar to the one used during training, which does not always represent a general performance level. Therefore, we argue that it is also the end user’s responsibility to generate evaluation data to assess the specific performance of any DL model for their data. This would often involve generating ground truth images or investing time in manually annotating a few images to ensure that sufficient material is available for this essential quality control step. For instance, when planning to use a denoising DL model, users can acquire a few corresponding high SNR images to ensure that the chosen denoising strategy works appropriately. Additionally, using such a dataset, users can also compare the performance of various tools to find the most suitable for the job (Figure 2B and 2C).

When comparing DL predictions to ground truth, it is important to visually assess the network output for artefacts, but equally important to quantitatively estimate similarity with the expected results. Box 1 presents a list of commonly used metrics and their appropriate uses depending on the tasks performed by the DL model. In addition, we provide a Jupyter notebook, as part of the ZeroCostDL4Mic platform ^22^, to easily compute these metrics directly in the cloud.

One of the most straightforward image metrics used to assess denoising, restoration, and image-to-image translation predictions is the Root Square Error (RSE), which calculates the sum of the square differences between predictions and the expected ground truth on a pixel-by-pixel basis. RSE is an easy-to-understand metric but does not report on structures, only on intensities. So other image similarity metrics such as the structural similarity index measure (SSIM ^38^) are also commonly used (Box 1 and Figure 2). Additionally, these metrics can be presented as maps that spatially render the discrepancies between the DL predictions and ground truth images. Such maps are especially useful to check for reconstruction artefacts that may be linked to specific structures in the images (Figure 2). Other metrics, such as Intersection over Union (loU), which measures the overlap between two binary masks, can assess the quality of segmentation outputs. Instance segmentation results can be further evaluated using additional scores such as F1 score or Panoptic quality ^37^, reflecting the ability of the algorithm to identify each object in the image correctly. Other metrics have also been developed to assess other image processing tasks such as image registration ^39^ or super-resolution reconstructions ^40^ but are not described here in detail.

When using metrics to assess DL predictions, an issue that often arises is to decide when the metric scores are good enough. This is often less of a problem for segmentation tasks where predictions and ground truth images can reach a good agreement (IoU and F1 scores of 0.9 and above). However, assessing the quality of denoising and image-to-image translation predictions may be more challenging. We found the approach of comparing both the prediction and the raw images to the ground truth images to be especially useful to evaluate denoising. This allows checking that the predictions are more similar to the ground truth images than the raw input data. If this is not the case, the DL model used is not improving the dataset toward the target image and should be reconsidered.

We recommend that efforts should be put into generating ground truth data as much as possible, and it is almost always possible to do so. But in rare cases, when ground truth images are not available, a careful visual inspection of the results may be the only option to assess a DL model’s performance. While less desirable, this solution may be sufficient if the results are already well characterised and well understood by the researcher such as when denoising known cellular structures. However, when studying novel phenomena, this approach should be avoided and observations cross-validated, especially if the structures observed after denoising are not easily visible in the raw data. Thus, there would be a need for developing metrics or novel evaluation methods that can assess the quality of predictions when no ground truth images are available.

## Choosing a DL tool

With the increasing availability of networks, models and software, it becomes challenging to identify the most suitable tool to answer a biological question. We do not recommend any particular software or tool simply because each user’s needs are distinct (for an excellent review of DL-based segmentation tools, see ^9^). Instead, we present a few pointers to help readers sieve through the literature based on what developers have reported in their work and reports from early adopters.

First, we recommend choosing an active, well-documented and well-maintained tool that matches the user’s prefered interface. Available DL tools now span various web interfaces ^29,32^, standalone software ^24,28,32,41^, plugins for popular image analysis software ^10,11,27,42^, online notebooks ^22^ and Python packages ^43^. Each platform requires a different level of technical skills to use. In addition, the details of the documentation provided by the developers can vary significantly and ranges from annotated code to online video tutorials and detailed step-by-step guides. This will limit accidental misuse of the tool and help the users understand the tools and their capabilities. Additionally, a substantial existing user base and online forums discussing troubleshooting are signs of a healthy and helpful tool. It also provides a wealth of information about users’ experiences as well as tips and tricks.

We advise being wary about works that do not provide source code and associated data for users to reproduce the results on example data. It is typically free and easy to make these publically available via common platforms (i.e. GitHub). We support works that themselves encourage open science. We also believe that example data are instrumental as they allow users to test and learn how to use a tool properly before applying it to their data.

As discussed above, it is essential to carefully assess the performance of DL-based tools on the dataset of interest. Therefore we also recommend using tools that offer purposely-built evaluation and sanity check strategies. We also strongly encourage users to consider how the chosen tool can be used within their prefered image analysis pipeline. DL-based analyses will often constitute only a small part of the overall analysis process, and therefore, the pipeline as a whole should be considered before selecting a tool.

When training DL networks using a new algorithm or software, one feature to look for is strategies to identify and prevent overfitting. Overfitting occurs when a model becomes too specialised to the training dataset and does not generalise well to new data. In practice, this means that the trained model may not perform well on new data even if they are similar to those used during training. Overfitting can be detected by monitoring how the performance of the model evolves over training time on the training dataset and a set-aside validation dataset. When more training leads to an improvement in performance on the training dataset but an otherwise worsening of the performance on the validation dataset, this is a sign that overfitting is occurring which can be typically visualised by plotting so-called loss curves over training time. Overfitting may be prevented by increasing the training dataset’s diversity using, for instance, data augmentation ^44,45^ or using strategies such as reducing the model complexity, adding regularisation (L1, L2) or early stopping during training ^46^. DL tools dedicated to training would enormously benefit from these features as these simplify the assessment and potential improvement on model optimisation for the user.

Another feature to look for when choosing a tool to train DL models is the possibility to perform transfer learning. Transfer learning enables the use of existing models as a starting point when training a new model. This allows taking advantage of previously learned model features present in these trained models instead of starting the training process from scratch. Transfer learning can considerably accelerate training or reduce the size of the necessary training dataset and produce models with higher performance ^22,47^.

Finally, when testing a new tool, it is often informative (and even often appreciated) to get in touch with developers and contribute to improving the tools when discovering bugs or by reporting issues in some particular configurations that may not have been encountered at the development stage. We feel the importance of this conversation is sometimes understated, even though it promotes good tools, open-mindedness and multidisciplinarity while building trust in the methods.

## Reporting the use of DL in publications

As previously done for other transformative technologies, we believe that the bioimaging community needs to discuss and flesh out guidelines for reporting DL use for bioimaging in publications ^48–51^. This is especially important as the reporting of more traditional image analyses and acquisitions pipelines is still raising concerns ^48,52–54^. It is beyond the intention of the present work to propose guidance to developers on evaluation and reporting when proposing new DL algorithms, and we refer the readers to recent work that has initiated this conversation within the computer science community ^55^. Instead, we focus on what would be useful to report when using DL tools.

Due to the wealth of hyperparameters, architecture choices and data manipulation available with DL, incorrectly trained or incorrectly evaluated DL models can be easily generated and lead to suboptimal results. This, therefore, highlights the importance of reporting clearly and appropriately the steps leading to the generation of a particular model. Indeed, standard guidelines will increase confidence in the use of DL and promote transparency and reproducibility. Such guidelines will also help reviewers assess manuscripts using DL for image analysis, especially if this technology is unfamiliar to them. Below, we listed several suggestions for contributing to this critical discussion.

- Naturally, the algorithm used should be reported, and the appropriate paper(s) cited. We also recommend indicating the version of the algorithm used or, failing that, the date at which the tool was obtained, since most analytical tools change over time, and each update may lead to varying performance on the same data. For DL, this is currently not a widespread habit, especially because both the network and the dataset may change over time (acquiring more data to expand the training dataset, for instance).
- Similarly, when using models trained by others, it is advisable to indicate the version of the model used. If not available, we recommend providing the date when the model was obtained and used.
- A DL model performance is entirely dependent on the dataset used at the training stage. When training dedicated DL models, the training dataset should be clearly described in the material and methods (types of microscopes, modality etc., as recommended in other work ^52^). Also, the training dataset should be deposited in a suitable and semi-permanent data repository (i.e. Zenodo, BioImageArchive).
- When training a DL model, we recommend indicating the key hyperparameters used and the main underlying libraries (e.g. TensorFlow, PyTorch). We recommend that DL models with reusability potential be deposited in a suitable repository (i.e. Zenodo) and linked to a model Zoo (i.e. TensorFlow hub, bioimage.io) along with their associated metadata.
- If custom code was generated to run the algorithm or process the data (pre or post-processing steps, for instance), it should also be shared with the paper and archived (i.e. GitHub, Zenodo).
- The steps taken to validate the DL model used should be clearly described. This includes the type of validation (i.e. indicating the evaluation metric used and what score was achieved), the number and the origin of the images used for evaluation (it is often considered imperative for evaluation data to be completely absent from training data to have bearings on how well the model generalises to new data), and explaining why the result was deemed acceptable. If space allows, we also recommend providing evaluation examples as supplementary figures.
- When performing predictions using a DL model, the tool used to run the model should be indicated (with the version again), and appropriate paper(s) cited. Indeed several tools offer the possibility to run DL models and may involve different pre-or post-processing steps that can influence the results obtained.

## Concluding remarks

DL tools are transforming the way we analyse microscopy images. However, we think that DL cannot be used on any dataset without prior validation. This is especially important as users risk falling into the artificial intelligence hype when other techniques may be more appropriate, more robust and sometimes quicker to analyse their images. Importantly, due to the complexity of operations performed in DL, not knowing precisely how the images are manipulated may affect how they can be reliably analysed downstream of DL. As an example, it is hard to estimate whether it is appropriate to quantify absolute image intensities following DL-based denoising due to potential non-linearity with respect to the input data. Similarly, although image-to-image translation and resolution improvement using DL are very promising approaches, they remain prone to undetected artefacts generation due to the inherent addition of data to the input data ^56^ from the training dataset, raising concerns of validity.

Here, we presented arguments towards the importance of validating any models using a purposefully-built evaluation dataset containing ground truth target images or labels. Similarly, the use of DL models should be reported appropriately to ensure reproducibility and transparency. This is a challenging task for DL since many components, both internal (hyperparameters) and external (training dataset) to the network used, can dramatically influence the results obtained. With the increasing availability of networks and models, we also stress the importance of finding ways to identify what might be a *good tool*. We believe that a good tool is not only a performant one, but that transparency of what it does to the data, useability and reliability are equally important. The responsibility of proper use of DL in microscopy is now equally shared between users and developers. Uncle Ben has never been more right than today: “With great powers comes great responsibility”. Finally, this article is not intended to set strict standards in place but rather serve as a starting point for further discussions between users, developers, image analysis specialists and journal editors to define appropriate use of these otherwise powerful techniques.

## Box 1 Common quality metrics used to assess denoising and segmentation DL models

All the metrics described here enable the comparison of the prediction generated by DL models to ground truth images or labels. Their respective use depends on the type of task performed by the DL model. In some cases, several metrics might be available and can be used together.

- **Image similarity metrics (denoising, restoration and image-to-image translation)**

Several metrics can be used to assess how similar two images are. These include:

1) **The Root Square Error (RSE)** map displays the root of the squared difference between two images. In this case, a smaller RSE is better. A perfect agreement between target and prediction will lead to an RSE map showing zeros everywhere.

$$RSE(i,j)=\sqrt{{\left(P(i,j)-GT(i,j)\right)}^{2}}$$

Where *P(i,j)* is the prediction value at pixel *(i,j)*, and *GT(i,j)* is the ground truth value at the same pixel. These images are typically normalised before evaluation of the metric.

2) **The normalised root mean squared error (NRMSE)** gives the average difference between all pixels in the images compared to each other. Good agreement between target and prediction yields low NRMSE values.

$$NRMSE=\sqrt{\frac{1}{N}\underset{i,j}{\sum^{\text{​}}}{\left(P(i,j)-GT(i,j)\right)}^{2}}$$

Where *N* is the total number of pixels, *P(i,j)* is the prediction value at pixel *(i,j)*, and *GT(i,j)* is the ground truth value at the same pixel. These images are typically normalised before evaluation of the metric.

3) **The Pearson correlation coefficient (PCC)** represents the degree of linear correlation between two images. A high correlation between target and prediction translates into a PCC close to 1.

4) **The structural similarity metric (SSIM)** evaluates whether two images contain the same structures based on contrast, luminance, and structural content concepts. It is a normalised metric, and an SSIM of 1 indicates a perfect similarity between the two images. The SSIM maps are generated by calculating the SSIM metric in each pixel but also considering the surrounding pixels. The **mSSIM** is the SSIM value calculated across the whole image ^38^.

5) **The Peak signal-to-noise ratio (PSNR)** is a metric that estimates the discrepancies between two images with respect to the peak signal amplitude of the prediction image. It is usually calculated in decibels, and the higher the score, the better the agreement.

- **Segmentation metric**

Image segmentation aims at defining areas of interest in an image based on their identity (foreground vs background being the most common one). A segmentation step typically provides a binary mask image where the pixels in the segmented area have a value of 1 (foreground) while the rest of the pixels have a value of 0 (background).

**The Intersection over Union (loU)** metric is a method that can be used to quantify the overlap between two binary masks. Therefore, when using IoU to assess the performance of a segmentation algorithm compared to ground truth masks, the closer to 1, the better the performance.

$$IoU=\frac{P\cap^{\text{​}}GT}{P\cup^{\text{​}}GT}$$

Where ∪ represent the union of 2 binary images (number of pixels that are foreground in either image) and ∩ represents the intersection of 2 binary images (number of pixels that are foreground in both images simultaneously). *P* is the predicted image, and *GT* the ground truth.

- **Instance segmentation metrics (also used for classification and object detection tasks)**

Instance segmentation aims to identify objects of interest in an image, both from the background and each other. An instance segmentation step commonly provides a label image where each identified object has a unique pixel intensity representing its identity, and the background is commonly set to have a pixel intensity of 0. Several metrics can be used to assess the quality of instance segmentation results, some of which are outlined below.

Typically, an IoU value is first calculated between the DL prediction and a GT image on a per-object basis. This allows identifying **true and false positives** as well as **false negatives**. True positives are objects that are correctly identified. In contrast, false positives are segmented objects that are not present in the ground truth image, and false negatives are objects missed by the segmentation algorithm. A particular object is considered as being detected when its segmentation mask has an IoU with the ground truth object mask that is above a user-defined threshold (for instance, IoU > 0.5). The number of false-positive (*Nb_False positive_*) and false-negative (*Nb_False negative_*) are then calculated as follow:

$$\begin{array}{c} {\mathrm{Nb}}_{\mathrm{False}\;\mathrm{positive}\;}=Nb_{\mathrm{Prediction}}-Nb_{\mathrm{True}\;\mathrm{positive}}\\ Nb_{\mathrm{False}\;\mathrm{negative}}=Nb_{\mathrm{GT}\;\mathrm{image}}-Nb_{\mathrm{True}\;\mathrm{positive}} \end{array}$$

*Nb_Prediction_* and *Nb_GT image_* refer to the number of objects present in the predicted image and the ground truth image, respectively.

**Precision** is defined as the number of correctly segmented objects divided by the total number of detected objects. Precision is a metric used to assess the cost associated with False Positives. The closer the precision is to 1, the better the performance.

$$\mathrm{Precision}\;=\frac{Nb\mathrm{True}\;\mathrm{positive}}{\mathrm{Nb}\;\mathrm{True}\;\mathrm{positive}+\mathrm{Nb}\;\mathrm{False}\;\mathrm{positive}}=\frac{Nb\mathrm{True}\;\mathrm{positive}}{Nb\mathrm{Prediction}}$$

**Recall** calculates how many of the actual positives the model captures by labelling them as True Positive. Recall can be used as a metric to assess the cost associated with False Negative. The closer Recall is to 1, the better the performance.

$$\mathrm{Recall}\;=\frac{\mathrm{Nb}\;\mathrm{True}\;\mathrm{positive}}{\mathrm{Nb}\;\mathrm{True}\;\mathrm{positive}+\;\mathrm{Nb}\;\mathrm{False}\;\mathrm{negative}}$$

**The F1** score combines both the precision and recall scores in a single metric and is calculated as follows.

$$F1=2\times \frac{\mathrm{Precision}\;\times \mathrm{Recall}\;}{\mathrm{Precision}\;+\mathrm{Recall}}$$

Other metrics such as **Accuracy** or **Panoptic Quality** (PQ, 1 indicates perfect agreement, ^37^) can also be used to score the quality of instance segmentation results.
